# PopNetD3—A Network-Based Web Resource for Exploring Population Structure

**DOI:** 10.1093/gbe/evz100

**Published:** 2019-05-16

**Authors:** Javi Zhang, John Parkinson

**Affiliations:** 1 Program in Molecular Medicine, Hospital for Sick Children, Peter Gilgan Center for Research and Learning, Toronto, Ontario, Canada; 2 Department of Biochemistry, University of Toronto, Ontario, Canada; 3 Department of Molecular Genetics, University of Toronto, Ontario, Canada; 4 Department of Computer Science, University of Toronto, Ontario, Canada

**Keywords:** population genomics, network visualization, chromosome painting, genetic relationships

## Abstract

We present PopNetD3, a web tool that provides an integrated approach for the network-based visualization of population structure based on the PopNet clustering framework. Users first submit a tab-delimited file that defines diversity of SNPs across the genome which is subsequently processed by the PopNet backend to define patterns of conservation at the chromosome level. The resulting population structure is visualized through a dedicated D3-based tool, allowing users to interactively examine chromosomal regions predicted to share ancestry. We illustrate the capabilities of PopNetD3 through an analysis of 16 strains of *Neisseria gonorrhoeae*. PopNetD3 is capable of processing population data sets consisting of hundreds of individuals and is publicly available online at: http://compsysbio.org/popnetd3 Last Accessed: May 17, 2019.

## Introduction

As the cost of sequencing continues to drop, there is increased interest in exploring population structure and the impact of strain diversity at the genome level (Narang et al. 1994; Skoglund et al. 2017; Gyorffy et al. 2018). Population software such as Neighbour-net, STRUCTURE, and Admixture (Pritchard et al. 2000; Bryant and Moulton 2003; Alexander et al. 2009) has been applied to investigate the population structure for many species of medical and agricultural importance (Su et al. 2012; Bedoya et al. 2017; Edea et al. 2017; Zhan and Zhu 2018). Significant improvements in predictive accuracy has been achieved (Falush et al. 2007; Hubisz et al. 2009), and innovative visualizations are now needed to aid the interpretation and application of the results.

We have previously developed PopNet, a population structure analysis tool with an innovative visualization scheme that integrates chromosome painting, a genome visualization approach previously described in fineStructure (Lawson et al. 2012), into a network-based framework (Zhang et al. 2017). Chromosome painting visualizes chromosomes as a bar composed of smaller segments, each representing a section of the genome, colored according to patterns of shared ancestry. Each bar is displayed as an annulus, allowing each individual to be represented as a node in a graph with colored segments indicating predicted recombination events. The additional dimension offered by networks, compared with hierarchical clustering or STRUCTURE-type block plots (Pritchard et al. 2000), provides greater resolution of genetic relationships between both individuals and subpopulations. Together, these features help the user interpret the results and understand their data.

To expand the accessibility of PopNet, we present PopNetD3, a web-based implementation of PopNet featuring cloud computing and an in-browser network visualization tool. PopNetD3 is accessible through any browser at www.compsysbio.org/popnetd3. The principle components are the job submission page, under the “Submit Job” tab, and the network visualizer under the “Visualization” tabs. The submission page allows the user to upload data files and parameters to be processed by PopNet on the cloud server. Upon completion, the user will be notified by email to view the results in the network visualizer. Within the visualizer, the user can retrieve jobs by a unique job id, view and manipulate the network, and download the network view as a PDF. Detailed documentation on the functions of PopNetD3 is provided through the “Tutorial” tab.

Compared with the standalone PopNet application, PopNetD3 improves accessibility, user interaction, and functionality. First, the user requirements are limited to access to a browser, with Google Chrome being recommended. Second, PopNetD3 features an interactive graphical interface, with updates being immediately accessible immediately. Finally, rather than relying on the Cytoscape framework (Shannon et al. 2003) used by PopNet, the D3-based (Bostock et al. 2011) network visualizer is tailored specifically for the visualization of PopNet generated networks.

In addition, the D3 visualization framework has led to the implementation of new features include group nodes, dynamic chromosome paintings, and an interactive view of aligned chromosomes. Group nodes can be used to quickly and neatly reshape the network to emphasize key patterns. Dynamic chromosome paintings allow the node-embedded chromosome paintings to be changed on-the-fly to focus on specific regions. Regions of interest can be examined in the aligned chromosome view to identify key genes and recombination events. The flexible framework is well-suited for continuous development and addition of features.

## Results

To demonstrate the capabilities of PopNetD3, we illustrate its application to 16 *Neisseria gonorrhoeae* genomes obtained from both sexes across several geographical settings. *Neisseriagonorrhoeae* is the causative agent of gonorrhea, which is estimated to infect 78 million new patients per year (WHO 2018). In addition to its ubiquitous presence, *N. gonorrhoeae* has developed resistance to many front-line antimicrobials currently used for its treatment (Day and Cole 2016). Previous studies have found *N. gonorrhoeae* to possess a nonclonal population structure with high rates of recombination (Didelot and Maiden 2010; Ezewudo et al. 2015). Compared with existing methods, PopNetD3 can better visualize inferred recombination events as well as their impact on the overall population structure.

**Fig. 1.**
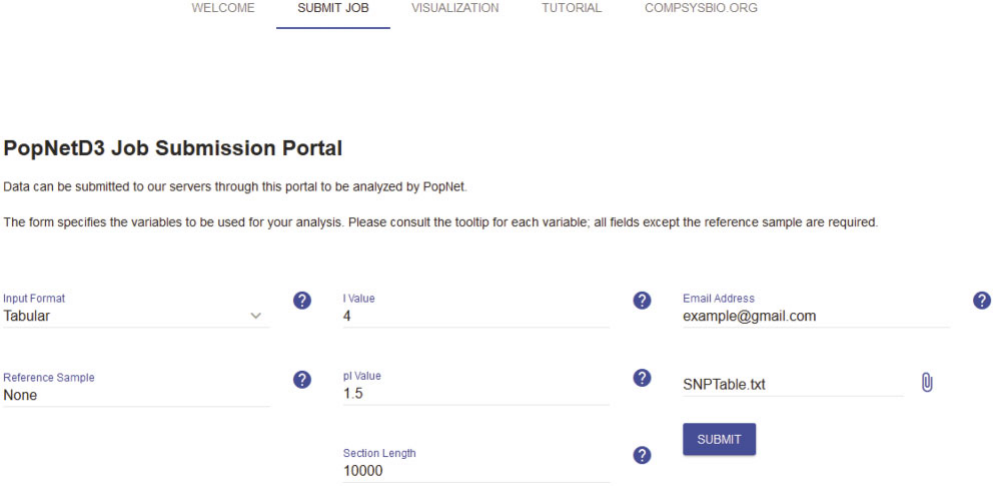
—The PopNetD3 job submission portal. The browser-based interface allows users to upload their own data to PopNetD3’s cloud server to be processes automatically. The data file should be a tab separated values (tsv) file containing whole genome SNPs from all samples. The key parameters are the I value, pI value, and section length, with recommended values of 4, 1.5, and 10,000, respectively. An email address is required to receive the job ID needed to view the resulting network and chromosome paintings.

The input file was created by aligning all samples to the FA1090 reference genome using MUMMER3.0 (Kurtz et al. 2004) and organizing the resulting SNPs into a tab-separated file (tsv). The file was uploaded to our server through the job submission portal, and subsequently processed by PopNet (fig. 1). The submission form includes several parameters required by PopNet. The species, input format, and reference sample parameters pertain to the input file. The species parameter refers to chromosomal naming conventions for certain species (i.e., names that are not strictly roman numerals). The reference sample parameter can be used when the first sample of the input file is the reference. A typical input not requiring these two considerations should select the default option for species and leave the reference sample blank.

The clustering inflation and pre-inflation parameters I and pI control the number of subpopulations defined by PopNet via MCL (von Dongen 2000). Higher I and pI values typically result in a more granular division of the population. The section length parameter controls the size of the chromosome sections used in chromosome painting. Shorter section length allows for more detailed chromosome paintings at the cost of decreased accuracy due to fewer SNPs within each section. The values *I* = 4, pI = 1.5, and section length = 5,000 are used for the *N. gonorrhoeae* data set presented here.

**Fig. 2.**
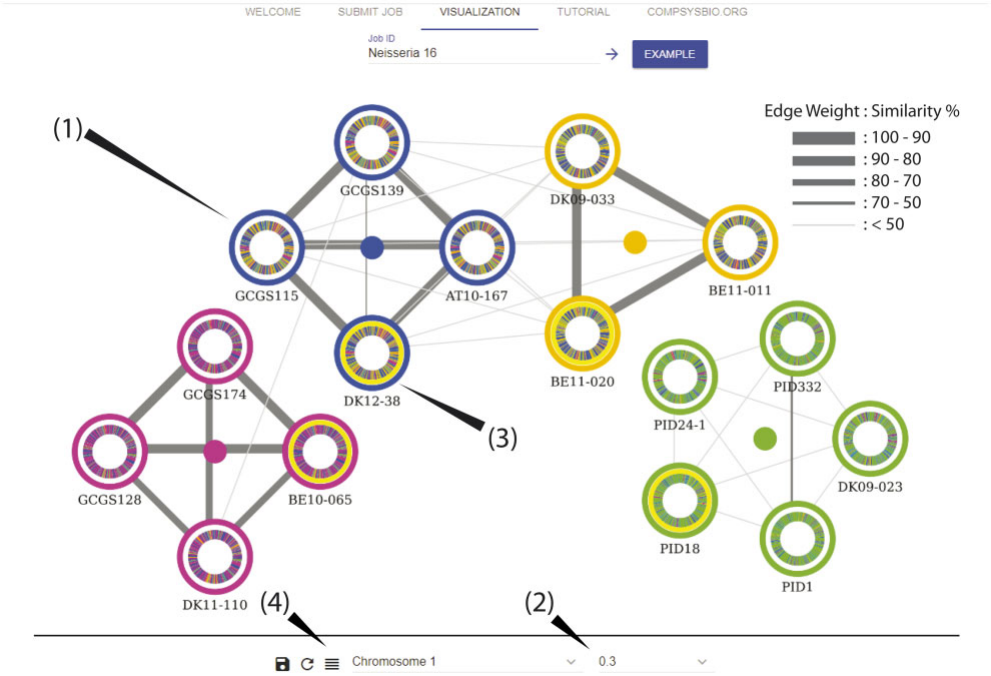
—The PopNetD3 network visualization tool showing a network of 16 *Neisseria gonorrhoeae* genomes consisting of four subpopulations as defined through PopNet analysis of SNP data. Each genome is represented by a single node (1), with border color indicating subpopulation membership. Edge width represents similarity of two genomes defined by the frequency of genomic sections that cocluster in the PopNet analysis. Edges between samples with <30% similarity were removed by selecting the cut off indicated (2). Circularized chromosome paintings of the sample are embedded within each node. Nodes can be selected and manually arranged (3). Multiple nodes can be selected to yield a detailed view of their chromosome clustering patterns (4).

The resulting network and chromosome paintings can be viewed through the in-browser network visualization tool. The network shows four subpopulations within the sample set, validated using hierarchical clustering (supplementary fig. 1, Supplementary Material online), each represented by a different color and positioned around a central “group node” (fig. 2(1)). Each node represents a single genome. Nodes can be repositioned through dragging, and groups can be moved together by dragging the “group node” in the center. The border color of a node indicates its subpopulation membership, and a chromosome painting of the sample is embedded within. Edges connecting each pair of nodes have widths corresponding to the degree of similarity as measured by coclustering frequency defined by PopNet. Edges between nodes with <50% similarity (i.e., cocluster in <50% of all genome sections) are hidden by default, but the cut-off can be changed dynamically (fig. 2(2)).

The chromosome paintings embedded within each node can be accessed in an aligned view after selecting a chromosome either on the control panel or by clicking on the corresponding section of the chromosome painting on any node. Nodes can then be selected or de-selected by mouse clicks (fig. 2(3)). Clicking the “Chromosome painting” button will display the chromosome paintings of all highlighted nodes (fig. 2(4)). Within the graph, each horizontal bar represents one genome, and is composed of smaller segments each corresponding to one section of the genome. The length (bp) of each section is determined by the “section length” parameter, set to 5,000 in this analysis. The color of each section indicates the predicted shared ancestry of that section. If the color is of the genome’s subpopulation, then no shared ancestry is predicted with any other subpopulation. Otherwise, the sample is predicted to share ancestry with the subpopulation corresponding to the color. Together, the network edges and chromosome paintings can be used to infer the genetic relationship between subpopulations as well as samples. The samples from male patients form three connected subpopulations: Magenta, blue, and yellow, whereas those from females form the green subpopulation. The clear separation observed suggest the presence of significant adaptation to host gender.

The thick edges connecting members of the magenta, blue, and yellow subpopulations indicate high intracluster homogeneity, where members cocluster in over 90% of their genomes. Thin edges connecting the subpopulations indicate lower relatedness between them, with samples across subpopulations coclustering in an average of 30% of their genomes. Of the three subpopulations, the blue and yellow subpopulations are closer to each other than with the magenta subpopulation, coclustering in 46% of their genome on average compared with 22% with the magenta subpopulation. Each subpopulation includes samples from both North America and Europe, indicating that geographic isolation is not observed in this population. In contrast, the green subpopulation containing samples from female hosts is relatively divergent, coclustering in 41% of their genome on average. With access to additional metadata such as patient symptoms or type of infection, standard statistical analyses can be used to find factors differentiating the male-patient subpopulations, as well as the source of divergence in the female subpopulation.

**Fig. 3.**
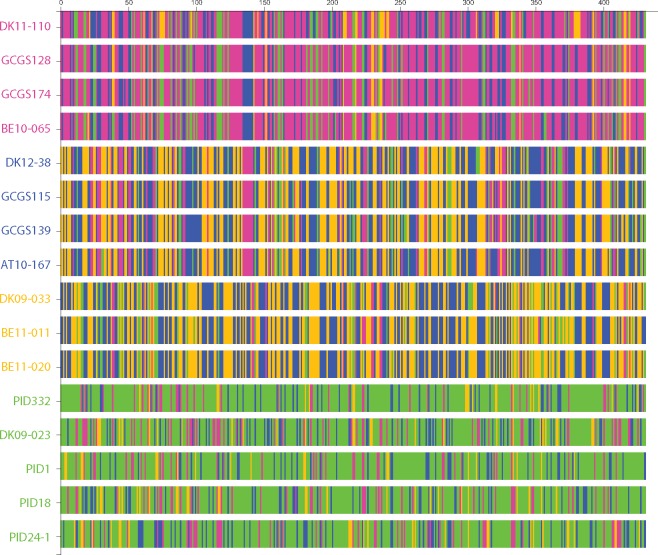
—Aligned chromosome painting of 16 *Neisseria gonorrhoeae* genomes reveal patterns of shared ancestry between each subpopulation. Using the PopNetD3 interface, individual strains can be selected from the network view and a more detailed view of their genetic relationships, as depicted through chromosome painting, generated by clicking on the chromosome view button (fig. 1(4)). Here, we show the chromosome paintings for the entire set of 16 strains depicted in figure 1. Each bar represents the genome of a single *N. gonorrhoeae* strain, divided into ∼430 5 kb segments (numbered along the top). Each segment is colored according to their predicted shared ancestry with other clades. Note how members of the blue, yellow, and pink subpopulations have similar chromosome paintings within each subpopulation whereas the green subpopulation shows significant variability between its members. The colored bar on the left show the PopNet subpopulation assignment.

The chromosome painting view can be used to confirm and expand upon observations from the network (fig. 3). Members of the homogenous subpopulations—magenta, blue, and yellow have similar chromosome paints with 90% identity on average. The more connected blue and yellow subpopulations contain many regions that are shared with each other, compared with the isolated green subpopulation which contains more regions attributed to itself. Chromosome paintings of blue subpopulation members contain on average 40% blue sections and 36% yellow sections. In comparison, members of the green subpopulation contain 69% green sections. Regions of shared ancestry between subpopulations tend to be short and dispersed throughout the genome, pointing to a model of short but frequent introgression between members of each subpopulation. Nonetheless, several significant regions of shared ancestry can be identified between each subpopulation. Despite their low overall similarity, the pink and blue subpopulations are predicted to share ancestry between 670 kb and 710 kb, a region containing eight enzymes including components of a Type I restriction modification (RM) system, which confers protection against infectious agents such as bacteriophages through degradation of foreign DNA (Loenen et al. 2014). Genes in regions of shared ancestry between subpopulations can serve as targets for drugs that aim to be broadly effective. Similarly, regions of shared ancestry within a subpopulation can point to the source of its unique properties. The samples labeled PID (corresponding to “*Pelvic Inflammatory Disease*”) of the green subpopulation share a region of common ancestry between 1,745 kb and 1,760 kb, which contains two genes related to the uptake of iron, a key factor regulating virulence in *N. gonorrhoeae* (Johnson 1985). Further investigation into this region may reveal PID-specific disease mechanisms.

## Discussion

The network and chromosome paintings offered by PopNet represent a novel and intuitive method for understanding the population structure of genome data sets. Compared with existing visualization methods, PopNetD3 offers three main advantages. First is the ability to view the population in different configurations, enabling quick and convenient comparisons between different subpopulations. As well, a different perspective can often expose hidden patterns in complex populations. Second is the ability to dynamically enrich the visualization with additional sources of data. Currently, only the circularized chromosome painting is implemented within each node, but the architecture of the visualizer allows for the incorporation of any data type to aid the generation of hypotheses concerning the evolutionary events that have helped shape population structure. Finally, the chromosome painting is a data source for downstream analysis, such as establishing association between genomic regions and phenotypic traits. At the same time, PopNetD3 remains flexible and scalable. As illustrated by the visualization of 173 strains of *Plasmodium falciparum* (supplementary fig. 2, Supplementary Material online), PopNetD3 can be used to visualize relationships across hundreds of individuals.

## Materials and Methods

### Input Files

PopNetD3 accepts SNP tables in the tab-delimited format (.tsv) as input. The table should contain one header row followed by one row per genomic position. The header row is composed of “#CHROM” and “POS” followed by the name of each sample. Subsequent rows are composed of the chromosome name, base pair position, and the genotype of each sample at that position. This table can be generated from the Genome Analysis Tool Kit (DePristo et al. 2011), or any SNP identification tool with postprocessing.

### Visualization Interface

PopNetD3 encompasses a browser-based interface, which includes a D3-based network visualizer, and a backend server. It is accessible at compsysbio.org/popnetd3. The network visualizer is a Scale Vector Graphics (SVG) object supported by a D3 force simulation engine (d3-force) for the display and manipulation of the network. The force simulation engine implements only collision forces, which prevent node overlap. Nodes, edges, and chromosome paintings within the network are derived from a JSON file generated by the PopNet pipeline hosted on the server. In addition, a group node is created for each subpopulation in the JSON file. By default, group nodes are place on the drawing area in a grid formation. Nodes are then placed in a circle around their respective group node. For larger subpopulations, nodes are placed in concentric circles instead. Individual nodes can be dragged to anywhere within the drawing area, whereas group nodes can be dragged to move the entire group.

In addition to the network visualizer, PopNetD3 provides a control bar allowing the user the following functionalities:

*Save*—Downloads a PDF of the current network to the user’s computer, named “PopNet.pdf.”

*Reset*—Returns all nodes to their starting positions.

*Chromosome painting*—Displays the aligned chromosome paintings of the selected nodes.

*Select Chromosome*—Changes the chromosome painting inside each node to that of the selected chromosome. The default setting, “all,” displays the concatenated paintings of all chromosomes.

*Edge Cutoff*—Hides edges between nodes with less than the specified similarity.

### Implementation

PopNetD3 is implemented in a combination of HTML, CSS, and JavaScript. The interface includes components from Material Design Lite and MDL-select. The network visualizer uses the D3.js, lodash, pdfkit, and jquery libraries. The server is implemented in JavaScript under the Node.js framework using the Express library. The body-parser library is used for communication between the browser and the server. The D3-node and svg2png libraries are used for server-side graphics rendering.

The server provides two main functionalities: Server-side computing and graphics rendering. Users may submit PopNetD3 job requests through the job submission portal. Upon receipt, the server will start a new instance of PopNetD3 using the uploaded data and parameters. Each job is assigned a unique ID. Upon completion, the server will send an email containing the job ID to the user supplied address. The server renders both the circularized and aligned chromosome paintings shown in the network visualizer. In both cases, SVG representations are created using D3-node, and then converted to PNG for improved performance.

## Supplementary Material

Supplementary data are available at *Genome Biology and Evolution* online.

## Supplementary Material

Supplementary_Material_evz100
