# Supplementary material for: PopNetD3—A Network-Based Web Resource for Exploring Population Structure
Source: Genome Biol Evol. 2019 May 16;11(7):1730–5. doi: 10.1093/gbe/evz100 (PMC6668580; doi:10.1093/gbe/evz100)
Supplement: Supplementary_Material_evz100 [file supplementary_material_evz100.pdf]

SUPPLEMENTAL FIGURES

Supplemental Figure 1. Subpopulations identified by PopNet are validated using hierarchical clustering.

PopNet uses overall co-clustering rates during its primary clustering step as a similarity metric to find subpopulations via Markov Clustering using MCL. Hierarchical clustering, under the Cluster 3.0 package (http://bonsai.hgc.jp/~mdehoon/software/cluster/software.htm#ctv), was applied to the same data used by MCL to validate its results. The settings used were uncentered Pearson correlation for similarity metric and centroid linkage for clustering method. Members of each subpopulation are shown to be closely related under hierarchical clustering, with each subpopulation forming a clearly identifiable group. Bar on the left show the PopNet subpopulation assignment.

Supplemental Figure 2. PopNetD3 network visualization of 173 strains of Plasmodium falciparum

To further illustrate the capabilities of PopNetD3, we present a view of 173 strains of P. falciparum presented in the original PopNet study (Zhang et al. 2017). In addition to the interactive web visualization (A), we provide a more detailed view generated through the interface as a PDF file. Each node represents an individual P. falciparum strain, with each of their 14 chromosomes divided by black lines. Edges weights represent similarity between connected nodes based on overall co-clustering frequency in PopNet. Only the top 20% of edges connected to each node, by weight, are shown. A moderate loading time (~1 minute) is initially required for visualizing relatively large networks or chromosome views, consisting of 100’s of strains, to allow the server

to generate each node/chromosome pattern. After loading, the interactive network visualizer allows nodes and node groups to be smoothly moved, even within such large networks. Note, additional nodes increase initial loading time but will otherwise have minimal impact on PopNetD3 performance.

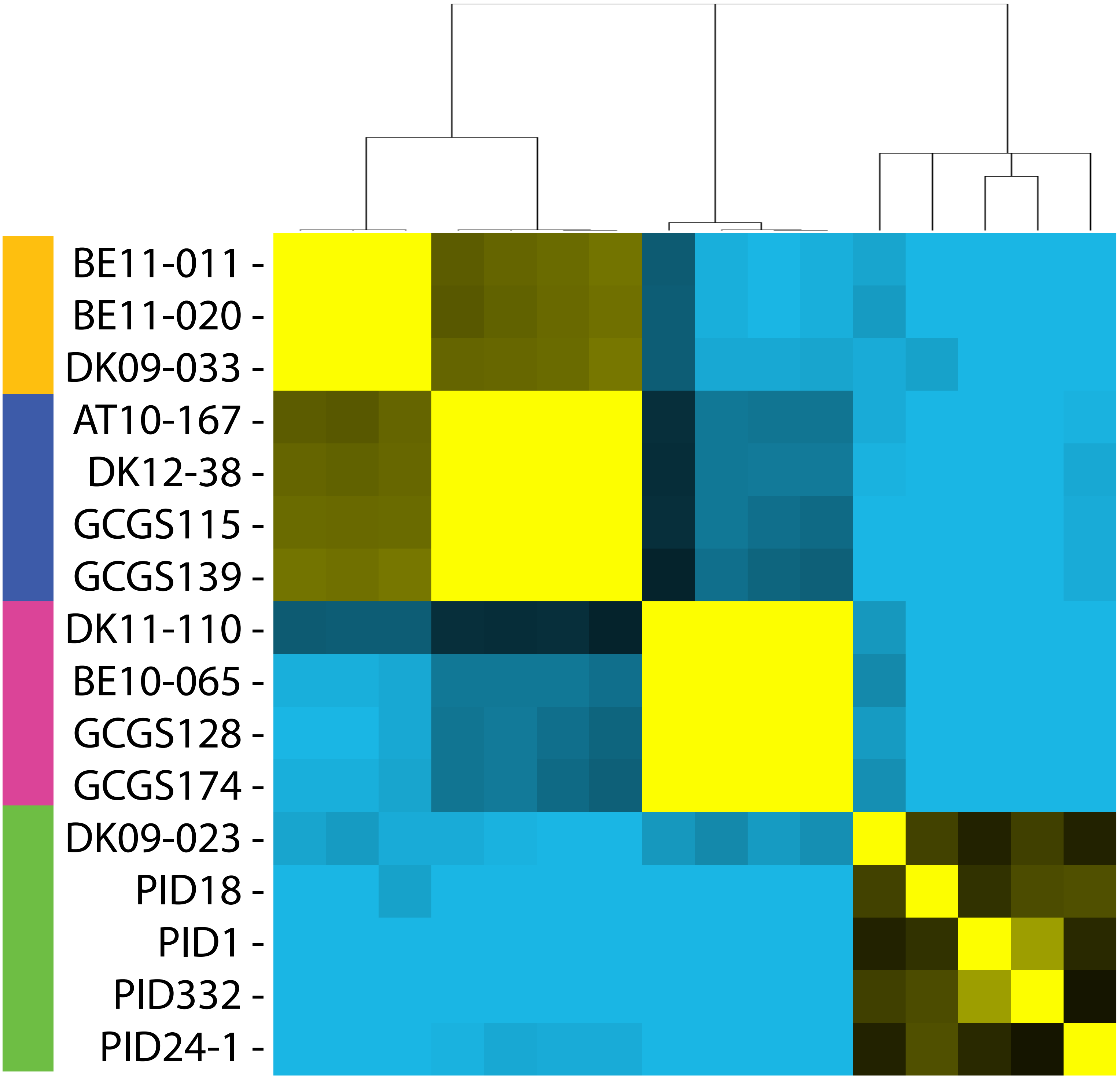
BE11-011
BE11-020
DK09-033
AT10-167
DK12-38
GCGS115
GCGS139
DK11-110
BE10-065
GCGS128
GCGS174
DK09-023
PID18
PID1
PID332
PID24-1

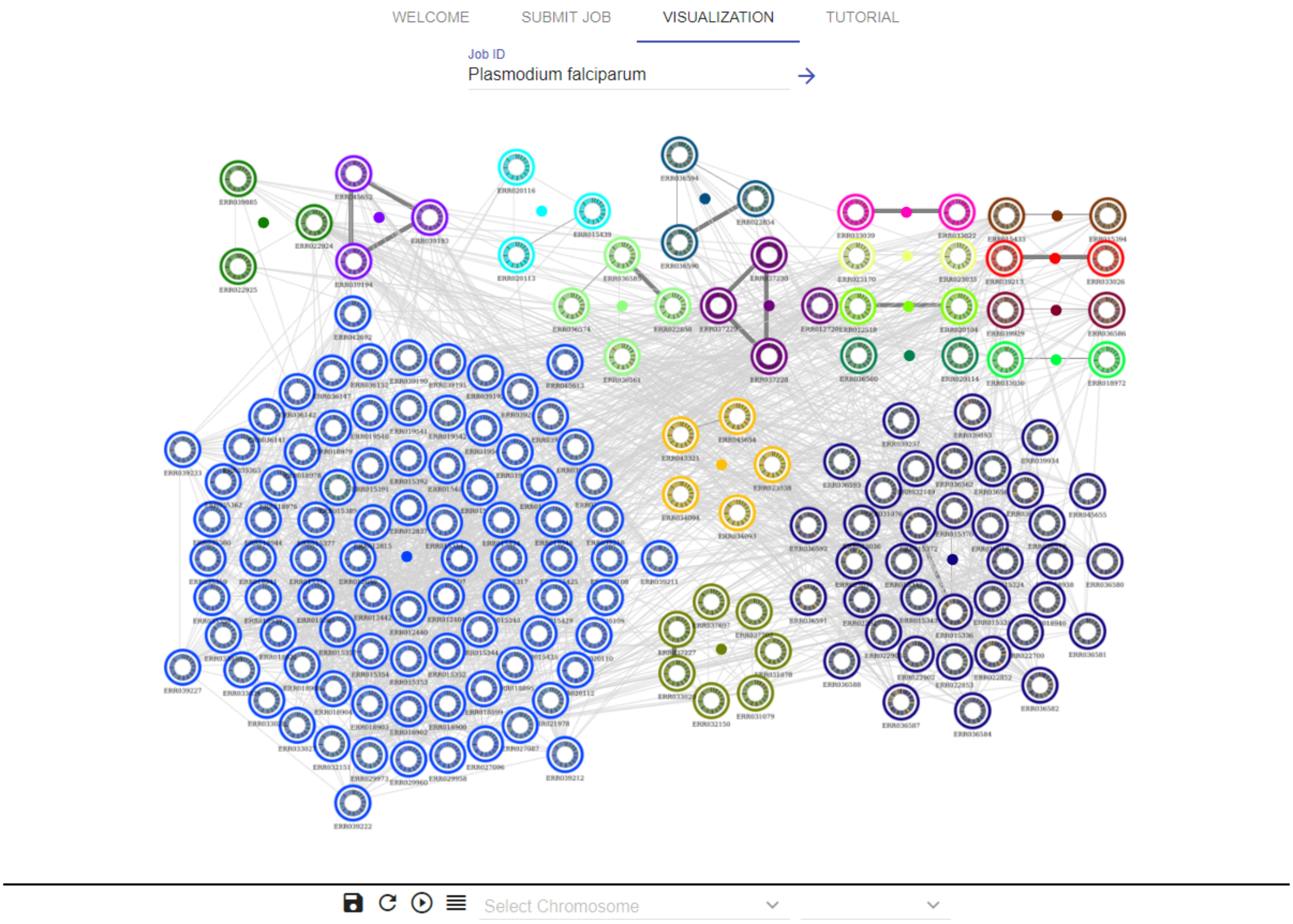
WELCOME
SUBMIT JOB
VISUALIZATION
TUTORIAL
Job ID
Plasmodium falciparum
Select Chromosome
